# Micro-dystrophin gene therapy prevents heart failure in an improved Duchenne muscular dystrophy cardiomyopathy mouse model

**DOI:** 10.1172/jci.insight.146511

**Published:** 2021-04-08

**Authors:** Zachary M. Howard, Lisa E. Dorn, Jeovanna Lowe, Megan D. Gertzen, Pierce Ciccone, Neha Rastogi, Guy L. Odom, Federica Accornero, Jeffrey S. Chamberlain, Jill A. Rafael-Fortney

**Affiliations:** 1 Department of Physiology & Cell Biology and Davis Heart and Lung Research Institute, College of Medicine, The Ohio State University, Columbus, Ohio, USA.; 2 Department of Neurology and Senator Paul D. Wellstone Muscular Dystrophy Specialized Research Center, University of Washington, Seattle, Washington, USA.

**Keywords:** Cardiology, Cardiovascular disease, Gene therapy, Mouse models

## Abstract

Gene replacement for Duchenne muscular dystrophy (DMD) with micro-dystrophins has entered clinical trials, but efficacy in preventing heart failure is unknown. Although most patients with DMD die from heart failure, cardiomyopathy is undetectable until the teens, so efficacy from trials in young boys will be unknown for a decade. Available DMD animal models were sufficient to demonstrate micro-dystrophin efficacy on earlier onset skeletal muscle pathology underlying loss of ambulation and respiratory insufficiency in patients. However, no mouse models progressed into heart failure, and dog models showed highly variable progression insufficient to evaluate efficacy of micro-dystrophin or other therapies on DMD heart failure. To overcome this barrier, we have generated the first DMD mouse model to our knowledge that reproducibly progresses into heart failure. This model shows cardiac inflammation and fibrosis occur prior to reduced function. Fibrosis does not continue to accumulate, but inflammation persists after function declines. We used this model to test micro-dystrophin gene therapy efficacy on heart failure prevention for the first time. Micro-dystrophin prevented declines in cardiac function and prohibited onset of inflammation and fibrosis. This model will allow identification of committed pathogenic steps to heart failure and testing of genetic and nongenetic therapies to optimize cardiac care for patients with DMD.

## Graphical abstract

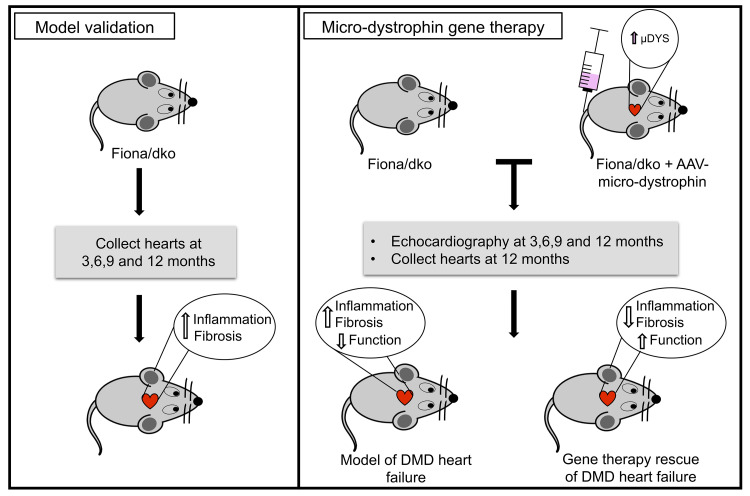

## Introduction

Duchenne muscular dystrophy (DMD) is an X-linked striated muscle degenerative disease resulting from loss-of-function mutations in the dystrophin gene (1). Milder Becker muscular dystrophy results from reduced size or levels of dystrophin, which acts to protect skeletal and cardiac muscle membranes by force distribution through interactions with filamentous actin and the dystrophin-glycoprotein complex (DGC) (2–4). Heart failure is the leading cause of death in both dystrophinopathies (1). Ongoing DMD gene therapy clinical trials with adeno-associated virus expressing an optimized miniaturized micro-dystrophin (AAV-μDys) suggest great promise for improving skeletal muscle pathology (5). However, it will be a decade before cardiac clinical outcomes are known since trials are being conducted in young boys and DMD cardiomyopathy emerges in the teens (3, 6).

Micro-dystrophins have repeatedly demonstrated efficacy on skeletal muscle pathology and function in preclinical models (7, 8). The impact of μDys on heart failure is unknown due to absence of a dystrophic model that reproducibly progresses into heart failure. Dystrophin-deficient *mdx* mice exhibit only a mild cardiomyopathy that never advances into reduced whole heart function (9–13). Dystrophin-deficient dogs exhibit a highly variable cardiac progression, making thorough evaluation of cardiac therapies impossible (14).

*Mdx* mice also deficient for the partially compensating dystrophin paralog utrophin (dystrophin/utrophin-deficient; dko) develop functional and histological clinical signs present at DMD cardiomyopathy onset (15–20). Dko mice show reduced cardiac muscle force that is detectable as left ventricular strain rate abnormalities, the earliest cardiac clinical sign in patients with DMD (18, 21). These force reductions occur concurrently with onset of cardiomyocyte degeneration but substantially precede reductions in whole heart function in mice and humans (15, 17, 18, 21). Cardiomyocytes become replaced with fibrosis, typically starting in the left ventricular epicardial free wall, prior to reduced whole heart function in dko mice and as detected by late gadolinium enhancement by cardiac magnetic resonance imaging (MRI) in DMD patients (16, 17, 20, 22).

Dko hearts represent a more severe cardiomyopathy model, but with the same underlying molecular mechanisms as *mdx* cardiomyopathy, with increases in only a small number of proinflammatory and profibrotic markers (19). Furthermore, transgenic expression of a mechanically functional dystrophin, but not dystrophins lacking the DGC- or actin-binding regions, are able to prevent cardiomyopathy onset, further supporting the relevance of dko cardiomyopathy to DMD (17). However, dko mice succumb to skeletal muscle pathology before developing heart failure, preventing therapeutic investigations on heart failure progression (23). To overcome these challenges, we have now developed a dystrophic dko mouse model with transgenic correction of skeletal muscles that progresses into late-stage heart failure over 12 months in a series of steps that parallels human DMD cardiomyopathy. We used this model to test efficacy of AAV-μDys gene therapy for the first time on the prevention of heart failure progression.

## Results

**Figure 1:**
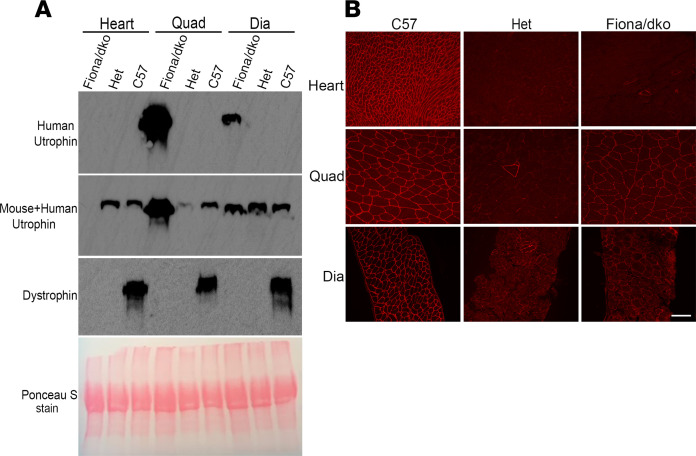
Validation of skeletal muscle-specific full-length utrophin expression in the Fiona/dko mouse model. (**A**) Western blot analysis of heart, quadriceps (Quad), and diaphragm (Dia) lysates obtained from Fiona/dko, utrn^+/–^ mdx (“Het”), and C57BL/10 (C57) wild-type control mice. Blots were incubated with antibodies specific for human utrophin (MANCHO7) (first panel) or to detect both mouse and human utrophin (MANCHO3) (second panel) and show full-length human utrophin protein is present in quadriceps and diaphragms, but not hearts, of Fiona/dko mice. Dystrophin (MANDYS1) (third panel) Western blot shows the presence of dystrophin only in wild-type C57 controls. Ponceau S staining (bottom) was utilized to verify equal protein loading. Blots shown are representative of a minimum of 6 blots run on at least 3 separate sets of biological replicates to ensure reproducibility. (**B**) Representative immunofluorescence images of heart (top), quadriceps (middle), and diaphragm (bottom) sections from C57, Het, and Fiona/dko mice stained with a polyclonal antibody that detects the C-terminus of both mouse and human dystrophin and utrophin. Technical duplicates of a minimum of 3 biological replicates were run for each experiment. Immunofluorescence staining shows human utrophin localization from the Fiona transgene in the pattern of wild-type dystrophin localization in Fiona/dko quadriceps and in a mosaic pattern in diaphragm but the absence of dystrophin localization or transgenic utrophin localization in Fiona/dko hearts. Low-level utrophin expression can be observed in Het tissues, and the presence of rare dystrophin-positive “revertant” myofibers and myocytes is also seen. Scale bar: 100 μm.

A full-length human utrophin transgene expressed from a skeletal muscle-restricted promoter/enhancer (Tg(ACTA1-Utrn)2^Ked^; “Fiona”) was used to rescue the skeletal muscle pathology of dystrophin/utrophin-deficient mice (*utrn^–/–^ mdx*; dko), allowing progression of cardiomyopathy (Fiona/dko) (24, 25). Fiona/dko mice showed high levels of utrophin expression in quadriceps muscles, and to a lesser extent in the diaphragm, but not in the heart (Figure 1A), as previously observed with the Fiona transgene on an *mdx* background (25). Transgenic utrophin was present in a pattern consistent with wild-type dystrophin localization inside the sarcolemma of skeletal muscle fibers, with no utrophin or dystrophin localization in hearts of Fiona/dko mice (Figure 1B). This expression pattern prevents dystrophic pathology and normalizes force in skeletal muscles (25) but allows progression of cardiac pathology in dko mice that otherwise succumb to skeletal muscle disease by 10–20 weeks of age (23, 24).

**Figure 2:**
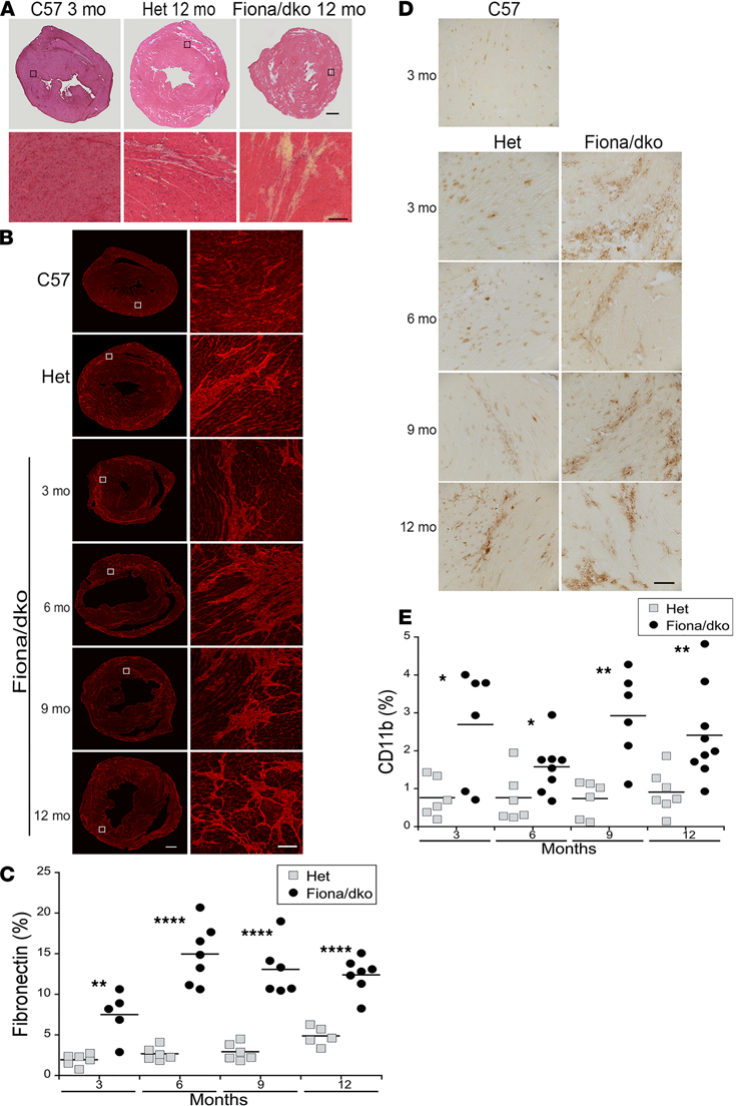
Cardiac fibrosis and inflammation is higher in Fiona/dko compared with Het mice. (**A**) Representative composite images of transverse heart sections through the ventricles from C57 wild-type controls and 12-month-old Het and Fiona/dko mice stained with hematoxylin and eosin (top) and zoomed images of left ventricular myocardium (bottom) corresponding to black boxes in the top panel. (**B**) Representative composite fibronectin immunofluorescence (red) images of heart sections from C57, Het (12 months), and Fiona/dko mice at 3, 6, 9, and 12 months of age (left). Zoomed images showing areas of scar formation corresponding to the areas boxed (white) in the left panels (right). Images shown are from samples near the mean for each age in **C**. (**C**) Quantification of fibrosis shown as percentage area of ventricular composite sections containing fibronectin staining (red). Percentage of fibronectin is higher in Fiona/dko compared with Het hearts at all time points, suggesting more scar formation in Fiona/dko mice. Statistical analysis performed with Student’s *t* test comparing groups at each time point (lines under *x* axes); **P ≤ 0.01 and ****P ≤ 0.0001. Dot plots display total *n* analyzed for each group at each age. (**D**) Representative images of CD11b immunohistochemical staining (brown) of myeloid immune cells in C57 and in 3-, 6-, 9-, and 12-month-old Het and Fiona/dko heart sections. Images shown are from samples near the mean for each age in **E**. (**E**) Quantification of CD11b myeloid cell infiltrate staining shown as percentage area of ventricular composite sections shows more inflammation in Fiona/dko compared with Het hearts at all time points. Statistical analysis performed with Student’s *t* test; *P ≤ 0.05 and **P ≤ 0.01. Dot plots display total *n* analyzed for each group at each age. Scale bars: 600 μm, composites; 100 μm, zoomed.

To characterize the cardiac phenotype in Fiona/dko mice, we compared fibrosis and inflammation, hallmark features of DMD hearts, with the previous *utrn^+/–^ mdx* (“Het”) cardiomyopathic mouse model. The Het model demonstrates clinical signs of cardiomyopathy onset, including fibrosis and reduced cardiac strain rate, and has been used for translation of cardiac therapies to patients with DMD, but does not progress into heart failure during its lifetime (26, 27). Hearts from Fiona/dko mice showed large crescent-shaped scarring patterns in the epicardial left ventricular free wall similar to those observed in dko mice and DMD patients (16) and displayed more replacement fibrosis throughout both ventricles, indicative of greater amounts of ventricular damage (Figure 2A). Quantification of fibronectin immunostaining showed higher levels of fibrosis as a percentage of ventricular area in Fiona/dko hearts between 3 and 6 months of age (7.48% ± 1.3% and 14.96% ± 1.37%), which then remained stable at 9 and 12 months of age (13.04% ± 1.34% and 12.37% ± 0.82%) (Figure 2, B and C). Fibrosis was significantly higher in Fiona/dko compared with Het hearts at 3, 6, 9, and 12 months (*P* = 0.0014, 0.0000059, 0.000029, and 0.000036, respectively, Student’s *t* test) with 1.94% ± 0.29%, 2.66% ± 0.34%, 2.90% ± 0.43%, and 4.85% ± 0.52% ventricular area in Het hearts (Figure 2C). Although small deposits of fibronectin can be present around vessels in normal hearts, this assay detected only 0.96% ± 0.12% of ventricular area in wild-type controls. Inflammation in Fiona/dko hearts, determined by quantification of immunostaining for the myeloid marker CD11b, was significantly higher at 3, 6, 9, and 12 months of age (*P* = 0.014, 0.0461, 0.0016, and 0.0092 respectively, Student’s *t* test) compared with Het mice, which possessed ventricular areas infiltrated by myeloid cells of 0.76% ± 0.21%, 0.76% ± 0.27%, 0.73% ± 0.19%, and 0.91% ± 0.21%, respectively (Figure 2, D and E). The presence of inflammatory cells remained high in Fiona/dko hearts, with 2.69% ± 0.61%, 1.57% ± 0.24%, 2.92% ± 0.47%, and 2.41% ± 0.40% of ventricular area infiltrated by myeloid cells at 3, 6, 9, and 12 months (Figure 2, D and E), despite no additional increases in fibrosis after 6 months (Figure 2C) and no quantifiable ongoing cardiomyocyte damage after 3 months (data not shown). Wild-type hearts showed 0.36% ± 0.11% of ventricular area containing resident myeloid cells.

Since no previous mouse model of DMD showed ejection fraction reductions consistent with heart failure (<45%), blinded longitudinal echocardiography was used to monitor function in hearts of Fiona/dko mice and Het/Fiona littermates and to evaluate efficacy of current therapies. Gene therapy clinical trials are underway with micro-dystrophins to replace the protein missing from striated muscles of patients with DMD. No dystrophic animal model previously existed that reproducibly progressed into heart failure, so the ability of micro-dystrophin delivered postnatally to prevent heart failure is unknown. Therefore, an additional group of Fiona/dko mice was treated intravenously at 4 weeks of age with 2 × 10^12^ vector genomes of AAV6-CK8e-Hinge3-micro-dystrophin (μDysH3, or μDys) (Table 1) (8, 28).

**Figure 3:**
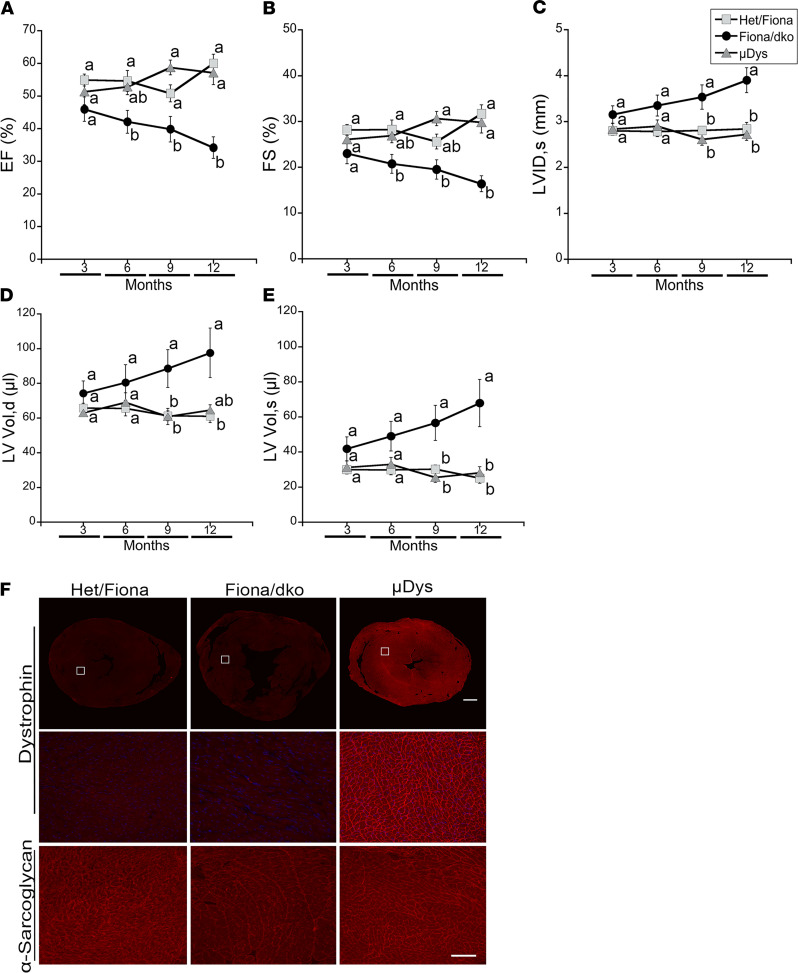
Fiona/dko mice show functional indicators of heart failure that are prevented by AAV-μDys treatment. (**A**–**E**) Longitudinal echocardiography measurements at 3, 6, 9, and 12 months of age in Het/Fiona, Fiona/dko, and μDys-treated Fiona/dko mice, including ejection fraction (EF) (**A**), fractional shortening (FS) (**B**), left ventricular internal diameter (LVID) at systole (**C**), and left ventricular volume (LV) at diastole (**D**) and systole (**E**). Fiona/dko mice showed significant reductions in function by 6 months of age and structural abnormalities by 9 months of age compared with Het/Fiona littermates. μDys cardiac expression prevented all functional and structural abnormalities in Fiona/dko mice throughout the 12-month study to the same extent observed in Het/Fiona hearts, which maintained cardiac function and did not progress into heart failure. Heart rates were maintained at an average of 450 bpm throughout echocardiography. One-way ANOVA followed by Bonferroni’s post hoc test for each parameter was performed to compare groups at each time point (lines under *x* axes). Means that do not share a letter are significantly different. Values are expressed as mean (markers) ± SEM (error bars). For **A**–**E**: *n* = 6 males/5 females for Het/Fiona; *n* = 6 males/5 females for Fiona/dko; *n* = 4 males/4 females for μDys groups. (**F**) Representative composite (top) and zoomed (middle) images of dystrophin immunofluorescence on transverse ventricular sections with an N-terminal dystrophin antibody that reacts to human and mouse dystrophin shows uniform dystrophin localization throughout both ventricles from AAV-μDys–treated Fiona/dko mice (μDys). Representative images from *n* = 8 of the μDys-treated group stained for quantification in Table 1. Members of the DGC, such as α-sarcoglycan (bottom), are only minimally destabilized in dko hearts, and localization is restored to normal in μDys-treated Fiona/dko hearts. Scale bars: 600 μm, composites; 100 μm, zoomed.

Similar to our previous studies with Het mice, hearts from Het/Fiona mice showed no reductions in ejection fraction (EF), reductions in fractional shortening (FS), or increases in left ventricular internal diameters or volumes over a 12-month period (26) (Figure 3, A–E, and Table 2). EF was maintained at normal levels from 55% ± 1.8% at 3 months to 60% ± 2.8% at 12 months in Het mice. In contrast, Fiona/dko mice showed highly reproducible reductions in EF and FS and larger left ventricular internal diameters at systole and volumes at diastole and systole compared with Het/Fiona mice (Figure 3, A–E). EF declined in Fiona/dko mice from 46% ± 3.7% to 42% ± 3.5% to 40% ± 3.9% to 34% ± 3.3% at 3, 6, 9, and 12 months of age and was significantly worse than Het/Fiona littermates at 6, 9, and 12 months of age (*P* = 0.1485, 0.0227, 0.0499, and <0.0001, respectively, Bonferroni’s test) (Figure 3A). Remarkably, μDys-treated Fiona/dko mice maintained entirely normal function, with an EF of 57% ± 3.6% at 12 months of age. EFs of µDys treated Fiona/dko mice were significantly improved over Fiona/dko at 9 and 12 months of age (*P* = 0.0012, 0.0001, Bonferroni’s test) (Figure 3A). μDys treatment also prevented reduced FS and higher left ventricular internal diameters and volumes in Fiona/dko hearts (Figure 3, B–E).

**Figure 4:**
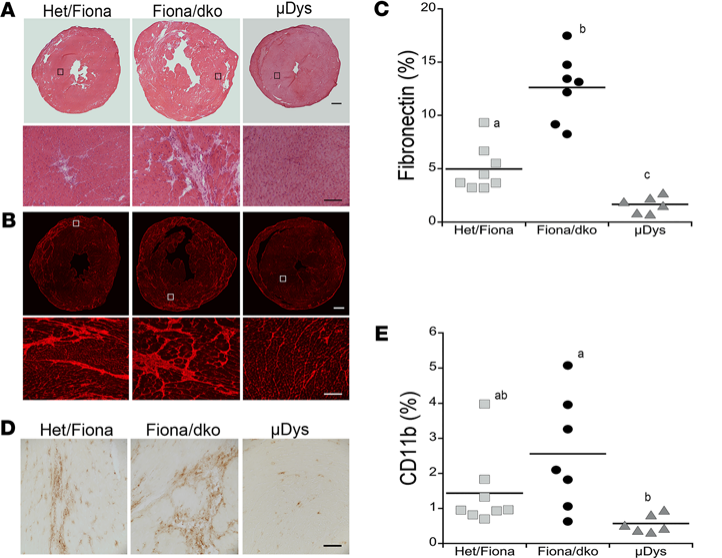
AAV-μDys treatment prevents cardiac fibrosis and inflammation in Fiona/dko mice. (**A**) Representative composite images of hematoxylin and eosin–stained transverse ventricular heart sections from Het/Fiona, Fiona/dko, and AAV-μDys–treated Fiona/dko (μDys) mice at 12 months of age (top). Zoomed images of boxed areas (bottom) demonstrate the absence of pathological features in μDys hearts. (**B**) Representative composite images of fibronectin immunofluorescence stained heart sections (top) demonstrate absence of replacement fibrosis in μDys compared with Het/Fiona and Fiona/dko mice at 12 months of age. Zoomed images show areas within white boxes in composites (bottom). Images shown are from samples near the mean for each group quantified in **C**. (**C**) Quantification of fibrosis shown as percentage area of ventricular composite sections containing fibronectin staining (red) demonstrates μDys prevents fibrosis through 12 months of age compared with both Het/Fiona and Fiona/dko hearts. Means for fibrosis quantification of Het/Fiona and Fiona/dko mice are well conserved with the separate cohorts of Het and Fiona/dko mice shown in Figure 1. Statistical analysis performed with Welch’s ANOVA (*P* < 0.0001) followed by Dunnett’s multiple-comparison post hoc test. Means that do not share a letter are significantly different. Dot plots display total *n* analyzed for each group at each age. (**D**) Representative images of CD11b immunohistochemical staining (brown) of myeloid immune cells in heart sections of 12-month-old Het/Fiona, Fiona/dko, and μDys mice. Images shown are from samples near the mean for each group quantified in **E**. (**E**) Quantification of CD11b staining demonstrates μDys prevents inflammation through 12 months of age compared with Fiona/dko hearts. Statistical analysis performed with Welch’s ANOVA (*P* < 0.0150) followed by Dunnett’s multiple-comparison post hoc test. Means that do not share a letter are significantly different. Dot plots display total *n* analyzed for each group at each age. Scale bars: 600 μm, composites; 100 μm, zoomed.

Cardiac pathology was present in dystrophin-deficient *mdx* and Het (Figures 2 and 3) models without a parallel decline in whole heart function. Inflammation and replacement fibrosis also preceded functional decline in Fiona/dko mice (Figures 2 and 3). Therefore, we next assessed whether μDys is sufficient to prevent cardiac pathology using hearts collected at 1 year of age from mice that underwent longitudinal echocardiography. μDys expression was present at cardiomyocyte membranes in 80% to 100% of ventricular area in all treated mice (Figure 3F and Table 1). Normal localization for α-sarcoglycan, the only dystrophin-glycoprotein complex member mildly reduced in dystrophic hearts (3), was restored by μDys (Figure 3F). Overall histology of hearts from Fiona/dko mice treated with μDys showed no signs of damage or scarring (Figure 4A). Remarkably, quantification of fibrosis in longitudinally echoed μDys-treated Fiona/dko mice showed dramatically lower percentages of ventricular area stained for fibronectin (1.68% ± 0.32%, *P* < 0.0001 Welch’s ANOVA) compared with untreated Fiona/dko (12.6% ± 1.2%, *P* < 0.0009 Dunnett’s test) and even Het/Fiona mice (5.0% ± 0.48%, *P* = 0.0088 Dunnett’s test) (Figure 4, B and C). Similarly, the total ventricular area containing myeloid cell infiltration was significantly lower (*P* = 0.0150 Welch’s ANOVA) in μDys-treated Fiona/dko hearts compared with untreated Fiona/dko hearts (0.57% ± 0.1% versus 2.56% ± 0.61%, *P* = 0.0480 Dunnett’s test), with Het hearts displaying an intermediate degree of inflammation (1.4% ± 0.4%, *P* = 0.1604, Dunnett’s test) (Figure 4, D and E).

## Discussion

This study demonstrated that Fiona/dko mice phenocopied DMD cardiomyopathy progression to heart failure (29) and represent an improved model that enables new understanding of committed pathogenic steps and allows testing of therapies to prevent, halt, or delay heart failure. We used this model to demonstrate for the first time to our knowledge that AAV-μDys postnatal delivery is efficacious at preventing heart failure progression and for completely preventing cardiac pathology. We previously showed dko mice phenocopy early-stage DMD cardiomyopathy, but cardiac studies of therapies in these mice were hampered by early mortality of the untreated controls (15–18, 26, 27). Studies with existing models led to identification of a long therapeutic window for cardiac interventions and facilitated design and translation of therapies that delay cardiomyopathy onset (26, 27). Nonetheless, there remained a critical need for a model that phenocopies DMD cardiomyopathy and progresses into heart failure, such that long-term efficacy of therapies in clinical translation can be evaluated. The stability of fibrosis during progression of cardiac dysfunction in Fiona/dko mice together with studies in old female *mdx* mice supports that fibrosis occurs prior to dysfunction and is nonreversible (30). Contributions of inflammation to heart failure can also be delineated in this model. These studies will be important for identifying the optimal therapeutic window in patients.

Despite a wealth of preclinical studies demonstrating AAV-μDys efficacy on dystrophic skeletal muscles, its ability to slow or prevent heart failure progression is not known. Even the earliest sign of cardiomyopathy, reduced ventricular strain rate, is not detectable in patients with DMD until ages 10–18 using state-of-the-art MRI (21, 31). Since ongoing gene therapy trials include boys as young as age 4, there was an urgency to evaluate maximal cardiac efficacy from AAV-μDys (5, 32). Previous studies of AAV-μDys were limited by lack of robust DMD cardiomyopathy models. Male *mdx* mice never progress to reduced EF, and *mdx* females do not develop functional cardiac deficits until 21 months of age, preventing any long-term assessment of therapies’ effects on heart failure progression (33). DBA-*mdx* mice do not show cardiac pathology comparable to DMD or altered function, and pathology in wild-type DBA controls makes this model unsuitable for cardiac studies (34).

We have previously demonstrated that dko hearts show the same underlying molecular and cellular mechanisms of cardiomyopathy as the *mdx* genotypic model of DMD. Expression of only 11 genes, in addition to utrophin, differ between *mdx* and dko hearts at cardiomyopathy onset, and 10 of these are in proinflammatory and profibrotic pathways known to be markers for human cardiomyopathy (19, 35). Expression of claudin-5, a tight junction protein in cardiovascular endothelia and at cardiomyocyte membranes, which is also reduced in the majority of failing human hearts, is the only other reduction in dko hearts (19, 36, 37). However, since transgenic expression of a mechanically functional dystrophin restores normal claudin-5 localization, this change occurs downstream from membrane instability and does not represent a distinct pathogenic mechanism resulting from utrophin absence, further justifying the validity of Fiona/dko mice as a DMD heart failure model (19).

The existing cardiac data for μDys function in early cardiomyopathy in *mdx* and dko mice show efficacy for diastolic dysfunction but not for very early signs of systolic dysfunction (9, 11–13). These studies are complicated by in vivo measurements with less-than-optimal heart rates, with minute deficiencies in early cardiomyopathy in a model with high variability, or requiring β-adrenergic stress to induce *mdx* cardiac dysfunction. The approach here focused on μDys efficacy on clinical measurements of systolic function, as this is the primary outcome for DMD heart failure. Inclusion of tissue Doppler measurements of diastolic dysfunction, a feature present in *mdx* mice and DMD patients, will be valuable in future work (13, 38, 39).

Rescue of skeletal muscles with a functional dystrophin transgene has been shown to exacerbate the onset of cardiomyopathy in *mdx* mice due to increased stress on the heart resulting from normal activity (40). The current study was performed at baseline in sedentary caged mice, so future studies investigating whether μDys is equally effective in exercised or stressed Fiona/dko mice are also warranted to best advise clinical cardiac care.

The Fiona/dko model will also be useful for future investigations to identify differences in pathological mechanisms between dystrophic heart and skeletal muscles. When dystrophin is absent from skeletal muscles, the entire DGC is destabilized from myofiber membranes (41). Mutations that result in loss of the DGC sarcoglycan components or altered glycosylation of the dystroglycan components lead to other forms of muscular dystrophy. However, in both dystrophin-deficient and dystrophin/utrophin-deficient hearts, the DGC remains localized to cardiomyocyte membranes, despite that mutations of some DGC members also lead to cardiomyopathy (17, 42). The cardiac dystrophin complex also contains additional interacting proteins Cavin-1 and Ahnak1, supporting at least some mechanistic differences underlying loss of membrane integrity in cardiac and skeletal muscles in DMD (2). Additionally, although neuronal nitric oxide synthase (nNOS) is localized by dystrophin to skeletal muscle membranes, nNOS does not localize to cardiomyocyte membranes (43, 44). nNOS has been shown to be reduced in dko hearts, but nNOS overexpression without membrane localization in dko mice reduces fibrosis in heart and skeletal muscles and slightly extends life span (3, 45). The stabilization of the DGC throughout heart failure evolution in Fiona/dko mice will allow future investigations to define underlying cellular pathological mechanisms involving dystrophin and its interacting proteins.

Several pharmacological therapies targeting different aspects of pathogenesis, including membrane instability, calcium dysregulation, reactive oxygen species accumulation, and mitochondrial dysfunction and energetics, have been shown to alleviate early dystrophic cardiomyopathy (3, 46). The availability of the Fiona/dko mouse model will enable testing these and other interventions at different steps in disease pathogenesis for their ability to prevent or halt progression to dystrophic heart failure.

## Methods

### Mice.

To generate dystrophin/utrophin dko mice (24) containing the human utrophin cDNA under control of the *Acta1* skeletal muscle-specific promoter (Tg (ACTA1-Utrn) 2^Ked^) (“Fiona”) (25), Fiona/*Dmd^mdx^* *Utrn*^+/–^ (Het/Fiona) mice were mated with *Dmd^mdx^* *Utrn*^+/–^ (Het) mice (24) to produce Fiona/*Dmd^mdx^* *Utrn*^–/–^ (Fiona/dko) at a frequency of 1 in 8 pups. The Fiona transgene was detected by PCR using primer sequences 5′-GTCAGGAGGGGCAAACCCGC-3′ (Utr_TG_For) and 5′-GTCGCTGCCCTTCTCGAGCC-3′ (Utr_TG_Rev). The knockout allele of utrophin was discriminated from the wild-type allele using 5′-GACAAACTGTCAGTTCTTAAG-3′ (UTRF1) and 5′-ACGAGACTAGTGAGACGTGC-3′ (NeoR) for detection of the utrophin knockout allele and 5′-GTGAAGGATGTCATGAAAG-3′ (PU65) and 5′-TGAAGTCCGAAAGAGATACC-3′ (Intron 7) for the wild-type allele (24). Both mouse lines have been previously backcrossed for many generations over decades with C57BL/10-*mdx* mice.

For Fiona/dko model validation, a cohort of Fiona/dko (*n* = 29 total, 15 males/14 females) and Het (*n* = 25 total, 14 males/11 females) littermates were dissected at 3, 6, 9, and 12 months of age. C57BL/10 mice (*n* = 8, 5 males/3 females ) were included as the wild-type controls but were obtained from separate matings bred in-house (obtained originally from Envigo). Heart, quadriceps, and diaphragm tissues were harvested for biochemical and histological analysis. A portion of each tissue was flash frozen in liquid nitrogen, and another portion was embedded in Optimal Cutting Temperature (OCT) medium (Sakura Finetek, 4583).

A separate cohort of Fiona/dko and Het/Fiona mice were used for AAV-μDys treatment, longitudinal echocardiography, and endpoint histological analysis as described below.

Personnel who completed data collection and analysis were blinded to the genotypes and treatment of the animals throughout the study.

### Western blot analysis.

Tissues from Fiona/dko, Het, and C57BL/10 quadriceps, diaphragm, and heart were homogenized and quantified using the *DC* Protein Assay (Bio-Rad, 5000166). One hundred micrograms of each sample was separated using SDS-PAGE, transferred to nitrocellulose, and incubated with monoclonal antibodies against dystrophin [MANDYS1(3B7), 1:5000, exon 31/32] or utrophin (MANCHO3, 1:100, crossreacts with mouse and human; or MANCHO7, 1:100, human specific) (University of Iowa Development Studies Hybridoma Bank). Primary antibody binding was detected with HRP-conjugated goat anti-mouse antibody (1:5000, Jackson ImmunoResearch Laboratories, 115035146) and developed using Pierce ECL Western Blotting Substrate (Thermo Fisher Scientific, 32106). Ponceau S staining (MilliporeSigma, P7170) was done for each Western blot as a normalizing control to ensure equal protein distribution between lanes. All Western blots were repeated with at least 3 biological replicates and at least 2 technical replicates for each to ensure reproducibility.

### Histopathology analyses and quantification.

Cryosections of 8 μm were stained with hematoxylin and eosin or antigen-specific antibodies. IgG immunostaining was performed using anti-mouse Alexa Fluor 488 (1:100, Invitrogen, Thermo Fisher Scientific, A11029) as previously described (47). Primary antibodies included a utrophin/dystrophin crossreacting rabbit polyclonal antibody (1:1000, Dystrophin C-term 18.4) (48), an affinity-purified rabbit polyclonal raised against the N-terminus of mouse dystrophin that is dystrophin specific but crossreacts with human dystrophin (1:100) (49), a rabbit anti-mouse fibronectin (1:40, Abcam, 23750), a rat anti-mouse CD11b (1:50, BD Pharmingen, 550282), or a rabbit anti-mouse α-sarcoglycan antibody (17). All histological quantification was conducted by an investigator blinded to genotype and treatment on *n* = 5–8 mice from each genotype for each time point for model validation and on 6–8 mice per genotype or treatment group from the longitudinal study group. All (*n* = 8) μDys-treated samples were used for dystrophin and α-sarcoglycan immunofluorescence. For quantification, transverse ventricular sections were imaged on a Nikon Eclipse 800 microscope under a 10× objective using a Nikon DS-Ri2 digital camera driven by Nikon NIS-Elements Br software and analyzed using Adobe Photoshop CS6 as previously described (50, 51).

### AAV-μDys production and treatment study design.

Design and construction of the μDysH3 (ΔH2-R23+H3/ΔCT) cDNA were previously described in detail (28). The AAV6-CK8e-Hinge3-micro-dystrophin vector was generated, produced, and titered as described previously (8, 52).

Fiona/dko mice at 4 weeks of age were randomly assigned into an untreated group (*n* = 11, 6 males/5 females) or a group receiving 2 × 10^12^ vector genome units of AAV6/CK8-Hinge3-micro-dystrophin (μDys) in 100 μL total volume normal saline delivered via tail vein injection (*n* = 8, 4 males/4 females). Untreated Fiona/dko, μDys-treated Fiona/dko, and untreated Het/Fiona mice (*n* = 11, 6 males/5 females) underwent longitudinal echocardiography at 3, 6, 9, and 12 months of age. No treated or untreated Fiona/dko mice died during the 12-month time course of the longitudinal echocardiography study. A few days after the 12-month echocardiography measurements, striated muscles were harvested and embedded in OCT as described above. Mice, hearts, and spleens were weighed and tibia length was measured.

### Echocardiography.

Echocardiographic measurements were taken using a Vevo3100 FUJIFILM VisualSonics system and MS-550 transducer by an investigator blinded to genotype and treatment. The mice were lightly anesthetized using 1.5% isoflurane (Piramal Critical Care, 6679401710), and parameters (Table 2) were determined in the M-mode using the parasternal short-axis view at the level of the papillary muscles. Measurements were calculated automatically using the Vevo LAB program, averaged from at least 3 consecutive systole-diastole cycles.

### Statistics.

Quantitative data are displayed as mean ± SEM. No data met predetermined exclusion criteria, and therefore all collected data were included in the analyses. The correct statistical test for each analysis was determined by first assessing the normality and variance of the data. For comparison of only 2 groups, Minitab software was used to determine equal or unequal variance, and a 2-tailed Student’s *t* test was performed taking into account either equal or unequal variance as appropriate for each data analysis. For longitudinal echocardiography measurements and calculations on groups in the AAV-μDys treatment study, a 2-way ANOVA was performed using Minitab statistical software to determine longitudinal changes in cardiac function (months of age), differences between groups, and their interaction effect. Since only group, but not months of age, showed a significant effect on each parameter using this analysis, more detailed analysis was then carried out using 1-way ANOVA followed by Bonferroni’s multiple-comparison post hoc tests using Minitab. A Welch’s ANOVA followed by Dunnett’s multiple-comparison post hoc test was used to compare fibronectin and CD11b quantification for the 3 groups in the treatment study using Prism version 8.4.3 statistical software (GraphPad). *P* ≤ 0.05 was considered significant.

### Study approval.

Animal protocols were approved by the IACUC of The Ohio State University, which is in compliance with the laws of the United States of America and conforms to the NIH *Guide for the Care and Use of Laboratory Animals* (National Academies Press, 2011).

## Author contributions

ZMH performed immunostaining and imaging, wrote Methods and figure legends, and edited the manuscript. LED performed blinded echocardiography and analysis. JL coordinated longitudinal study, dissected mice and cut cryosections, performed statistical analysis, and generated figures. The following was considered in determining the order of co–first authors: ZMH is a graduate student and JL is a research associate in the senior author’s lab; since LED works in a coauthor’s lab on an unrelated project and was blinded for this project, ZMH provided intellectual input on design and interpretation of this project. MDG maintained the mouse colony; genotyped, enrolled, and dissected mice; performed immunostaining; and drafted Methods. PC maintained and genotyped the mouse colony, assisted with Western blots, cut cryosections, performed immunostaining, and drafted Methods. NR performed immunostaining, Western blots, and blinded quantification of histopathology. GLO designed and generated μDys vectors and edited the manuscript. FA supervised and reviewed echocardiography data and edited the manuscript. JSC designed and generated AAV-μDys and treatment strategy, reviewed final data, and edited the manuscript. JARF conceived, designed, and supervised the overall study; reviewed primary data and analysis; and drafted portions of the manuscript.

## Figures and Tables

**Table 1:**
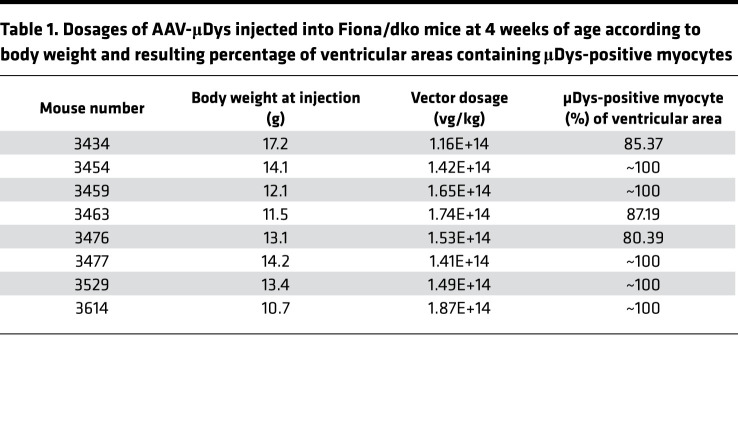
Dosages of AAV-μDys injected into Fiona/dko mice at 4 weeks of age according to body weight and resulting percentage of ventricular areas containing μDys-positive myocytes

**Table 2:**
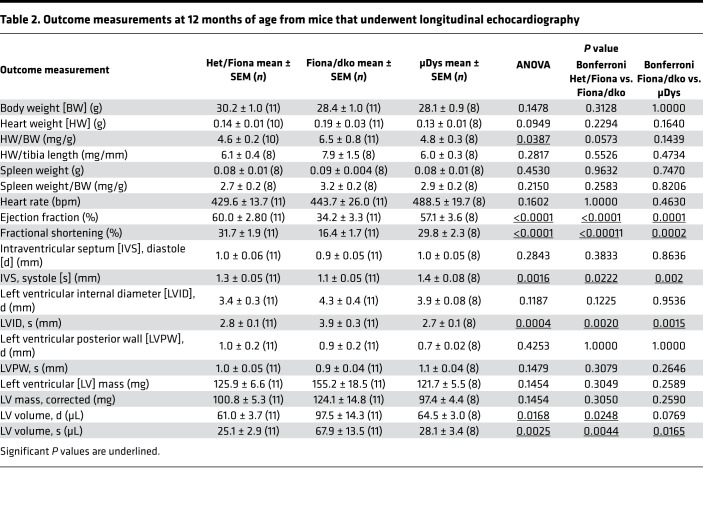
Outcome measurements at 12 months of age from mice that underwent longitudinal echocardiography

## Version 1 03/02/2021

In-Press Preview

## Version 2 04/08/2021

Electronic publication
